# Toxicity of Thiamethoxam Against Philippine Subterranean Termites

**DOI:** 10.1673/031.007.2601

**Published:** 2007-04-19

**Authors:** Menandro N. Acda

**Affiliations:** Department of Forest Products and Paper Science, College of Forestry and Natural Sciences, University of the Philippines Los Banos, College, Laguna 4031, Philippines

**Keywords:** Thiamethoxam, Macrotermes gilvus, Nasutitermes luzonicus, Microcerotermes losbanosensis, Termitidae, subterranean termites

## Abstract

Thiamethoxam (ACTARA^®^ 25WG) was evaluated for its termiticidal properties against three species of economically important subterranean termites (Isoptera: Termitidae) in the Philippines: *Nasutitermes luzonicus* Oshima, *Macrotermes gilvus* Hagen, and *Microcerotermes losbanosensis* Oshima. Results of the study indicated that exposure to soil or ingestion of paper treated with thiamethoxam at concentration above 0.41 ppm may provide an adequate chemical barrier or induce high mortality against *N. luzonicus*, *M. gilvus* and *M. losbanosensis* after 5–9 days. Feeding bioassays showed that thiamethoxam was not repellent to *M. gilvus* and *M. losbanosensis* but had an anti-feeding effect on *N. luzonicus*.

## Introduction

Termites (Isoptera: Termitidae) commonly occur in tropical soils, especially in the rain forest, where they play an important part in soil ecology by recycling wood and decaying plant materials (Lee and Wood 1971). Unfortunately, they become economic pests when their appetite for wood extends to human homes, timber structures and agricultural crops. The Philippines supports a rich termite fauna comprising of fifty-five known species in eighteen genera (Snyder and Francia 1963; Acda 2006). Of the 55 reported species, four subterranean species are considered serious structural pests; *Coptotermes vastator* Light, *Nasutitermes luzonicus* Oshima, *Macrotermes gilvus* Hagen, and *Microcerotermes losbanosensis* Oshima. These species are widely distributed and are considered serious structural pest of timber structures (Valino 1967; Acda 2004). The total damage caused by these termites in the Philippines is unknown but considering the abundance and level of activity of these insects, financial losses due to termite attacks are large (Acda 2004).

In recent years, the use of baiting techniques (Su 1994; Tsunoda et al. 1998; Getty et al. 2000, Sajap et al. 2000; Evans 2001) and non-repellent insecticides (Kard et al. 1989; Boucias et al. 1996; Potter and Hillery 2002) have resulted in successful control or elimination of invading termite colonies. However, the ability of termite baits and non-repellent chemicals to control higher species of subterranean termites (Termitidae) under tropical conditions is still unclear. At the moment, there is no method that can rapidly and effectively destroy subterranean termite colonies common in the tropics.

Thiamethoxam is a new neonicotinoid insecticide with stomach and contact activity (Yamamoto 1996; Mason et al. 2000, Meienfisch et al. 2001). It interferes with the nicotinic acetylcholine receptor, thereby disrupting the activity of the central nervous system and causing death to the insect. Thiamethoxam has a wide spectrum of activity against aphids, whiteflies and leafhoppers (Senn et al. 1998). It has also been reported to be non-repellent and has anti-feeding action against higher species of African termites (Delgarde and Lefevre 2002a). Recent studies showed that thiamethoxam was toxic and provided effective barrier against the Formosan subterranean termite (*Coptotermes formosanus* Shiraki) and the eastern subterranean termite (*Reticulitermes flavipes* Kollar) (Remmen and Su 2005A, 2005b). In this study, the termiticidal properties of thiamethoxam were investigated against three economically important species of subterranean termites (Termitidae) in the Philippines; *N. luzonicus, M. gilvus*, and *M. losbanosensis*.

## Materials and Methods

### Termites

Termites from three active field colonies of *N. luzonicus, M. gilvus* and *M. losbanosensis* located in the University of the Philippines Los Banos campus were collected by breaking and carefully tapping infested logs or secondary nest materials into plastic trays containing moist paper towels. Termites were then immediately transported to the laboratory and placed inside 100 liter plastic containers with lids and kept in a room at 25°C. Distilled water was sprayed on the inside walls of the container to keep the relative humidity above 80%. Mature worker and soldier termites were separated from logs or nest debris by breaking and sharply tapping materials into plastic trays containing moist paper towels. Termites were then sorted using a soft bird feather and used for bioassay within one hour of extraction and segregation. Mean body weight of workers for each species was determined by weighing 5 groups of 10 termites from each colony.

### Thiamethoxam

Thiamethoxam, 3-(2chloro-thiazol-5-ylmethyl)-5-methyl-{1,3,5}oxadiazinan-4-yldene-N-nitroamine, was provided by Syngenta Philippines, Inc. (Makati City, Philippines). The formulation used (ACTARA^®^ 25WG) contains 250 g (AI) per kg of product. Formulated granules were mixed in distilled water to achieve a suspension containing 410 ppm active ingredient (a.i.) concentration. The concentration of the suspension was determined based on the solubility of thiamethoxam in water at 25°C (Rust et al. 2004). After non-soluble material settled, serial dilutions were made of the supernatant to obtain 0.041, 0.41, 4.10 and 41 ppm thiamethoxam solution. Concentrations were selected based upon preliminary tests.

### Toxicity Test

Toxicity tests were performed to determine the ability of thiamethoxam to kill *N. luzonicus, M. gilvus* and *M. losbanosensis* by contact or ingestion of treated material. One hundred workers plus 10 soldiers of *N. luzonicus, M. losbanosensis* or fifty large workers plus 5 large soldiers of *M. gilvus* were placed in Petri dishes (9 cm diameter by 1.5 cm high) containing 15 g sifted oven dried, loamy soil (100 mesh) treated with ACTARA^®^ 25WG solution. Petri dishes containing treated soil were allowed to stand in a fume hood for 24 hours before the introduction of termites. Concentrations tested were 0.041, 0.41, 4.10 and 41 ppm of thiamethoxam in the soil (weight [AI]/weight of soil). Moist filter paper (Whatman #1) measuring 4 cm by 4 cm was added to serve as food. Petri dishes containing untreated oven dried soil moistened with distilled water and filter paper as described above were used as control. Experimental units containing *N. luzonicus* or *M. losbanosensis* were placed in an incubator maintained at 28°C and 85% relative humidity and exposed to treated soil for 14 days. Petri dishes containing *M. gilvus* were placed in plastic boxes (10 × 25 × 32 cm) with lid containing a layer (2.5 cm) of wet sawdust at the bottom to keep the humidity close to 100% and kept in an incubator maintained at 30°C for 7 days. Different conditions and period of exposure was used for *M. gilvus* due to high natural mortality below 85% relative humidity and prolonged laboratory assay conditions. After the prescribed exposure period, percent mortality was determined by examining the experimental units for dead termites. Workers were considered moribund when they no longer walk or stand when probed with forceps. Mortalities were corrected by Abbott's formula (1925) and lethal concentrations (LC_50_ and LC_90_) calculated by probit analysis (SAS Institute 1998). The test was replicated three times for each colony with a total of nine replicates for each concentration. The 95% confidence limits were calculated and used for comparisons of toxicities to thiamethoxam between termite species.

**Table 1.**
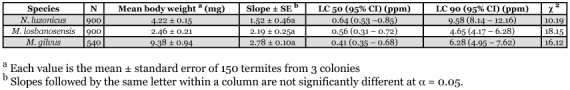
Toxicity of thiamethoxam against *Nasutitermes luzonicus, Macrotermes gilvusand Microcerotermes losbanosensis* after 1-week exposure to treated soil.

**Table 2.**
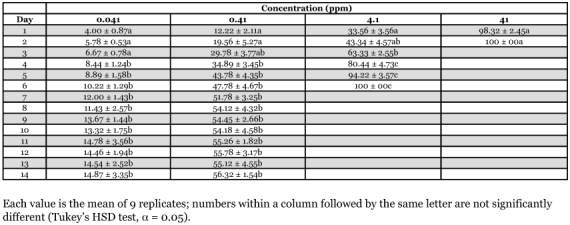
Daily mortality (% ± SE) of *Nasutitermes luzonicusworkers* after exposure to filter paper treated with thiamethoxam for 24 hours

A no-choice feeding bioassay was performed to determine trends in mortality of *N. luzonicus, M. gilvus* and *M. losbanosensis* upon ingestion of filter paper treated with various concentration of thiamethoxam. The bioassay was conducted using Petri dishes (9 cm in diameter) containing 15 g sifted oven dried soil wetted with distilled water and a 4 × 4 cm Whatman #1 filter paper impregnated with ACTARA 25WG solution to give 0.041, 0.41, 4.10 and 41 ppm thiamethoxam on paper (weight [AI]/weight of paper). Treated filer papers were allowed to stand in the fume hood for 24 hours before the introduction of termites. Termites fed with filter paper wetted with distilled water served as the control. One hundred workers plus 10 soldiers of *N. luzonicus, M. losbanosensis* or 50 large workers plus 5 large soldiers of *M. gilvus* were placed in each dish. Petri dishes containing the termites and filter paper were then placed in an incubator or plastic tray as described above. Termites were force-fed on the treated paper for 24 hours and then transferred to similar petri dishes containing untreated filter paper. Dead or moribund workers were recorded and removed from each unit daily. Workers were considered moribund when they no longer walk or stand when probed with forceps. Percent mortality was corrected using Abbott's formula (1925), arcsine square root transformed and then subjected to an analysis of variance (ANOVA) using a completely randomized design. The means were separated for each concentration using Tukey's Honest Significant Difference (HSD) test (α = 0.05, Statgraphics 1999). The test was replicated three times for each colony with a total of nine replicates for each concentration.

**Table 3.**
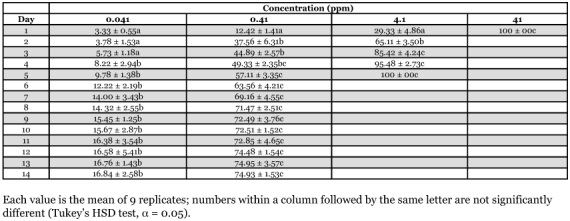
Daily mortality (% ± SE) of *Microcerotermes* losbanosensisworkers after exposure to filter paper treated with thiamethoxam for 24 hours

## Results and Discussion

The lethal concentration of thiamethoxam varies among the three species of subterranean termites tested (Table 1). Based on their respective LC_90_ and mean body weight after 1 week of exposure, thiamethoxam was about 2.86–3.43 times more toxic to *M. gilvus* than to *M. losbanosensis* or *N. luzonicus*. The slope for LC_50_ and LC_90_ for all three species did not overlap indicating a significant difference in toxicity for the species tested. Time to attain >*50%* and >*90%* mortality was 5–9 and 7–12 days of exposure, respectively, for all three species. These values are close to those reported for higher species of African and Brazilian termites (Delgarde and Lefevre 2002a, 2002b) and for imidaclopid, a first generation neonicotinoid compound closely related to thiamethoxam (Gahlhoff and Koehler 2001), but higher than those reported for *Coptotermes formosanus* and *Reticulitermes flavipes* (Remmen and Su 2005a). These results suggest a difference in thiamethoxam resistance between higher and lower species of termites. A significant decrease in tunneling activity and lethargy followed by death were observed in termites exposed to treated paper. Thiamethoxam acts at nicotinic acetylcholine receptors in the insect nervous system disrupting its normal functions, which could explain the above observations (Yamamoto 1996; Mason et al. 2000; Meienfisch et al. 2001). Results of the contact toxicity suggest that the sensitivity of higher species of termite belonging to the same subfamily may vary from species to species.

**Table 4.**
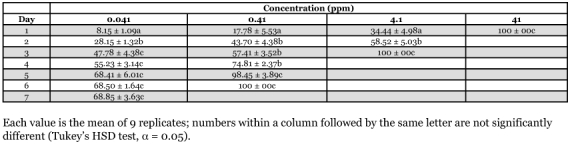
Daily mortality (9% ± SE) of *Macrotermes gilvus* workers after exposure to filter paper treated with thiamethoxam for 24 hours

Trends in mortality upon ingestion of filter paper treated thiamethoxam showed that paper containing >0.41 ppm resulted in high mortalities (>50%) in workers of *M. losbanosensis* and *M. gilvus* after 4–7 days (Tables 2 and 3). Similar results were observed with *M. gilvus* at concentration as low 0.041 ppm in 4 days (Table 4). Treated papers were consumed to various degrees by both *M. losbanosensis* and *M. gilvus* suggesting that the chemical was non repellent to these species. However, workers of *N. luzonicus* consumed very little of the treated paper at all concentrations tested. The amount of treated paper consumed was not determined in this study. Delgarde and Lefevre (2002a) showed that mortality in feeding and deterrence tests of African termites was not correlated with the quantity of thiamethoxam ingested. This would suggest that thiamethoxam was repellent or has anti-feeding effect on *N. luzonicus* and the high mortality in the feeding bioassay may be due to contact with the chemical. Delgarde and Lefevre (2002a) observed similar behavior with *Trinervitermes trinervius* Rambur (Nasutiterminae) and *Amitermes evuncifer* Silvestri (Termitinae). The above results with *N. luzonicus* could be problematic if this material were used as active ingredient in a termite bait product.

This study indicated that soil treated with thiamethoxam at concentration >0.41 ppm may provide an adequate chemical barrier against *N. luzonicus, M. gilvus* and *M. losbanosensis*. Lower concentrations of thiamethoxam (<0.041 ppm) may also be feasible for *M. gilvus*. The feeding bioassay indicated that ingestion of paper impregnated with >0.41 ppm thiamethoxam can induce high mortalities in the three species tested. *M. gilvus* and *M. losbanosensis* consumed treated paper to various degrees suggesting that the chemical was not repellent to these species. However, *N. luzonicus* may be repelled by the presence of thiamethoxam resulting in reduced feeding of treated paper. This would suggest that the high mortality of *N. luzonicus* during the feeding tests may be due to contact with the chemical as earlier reported.
